# Development, progress and future prospects in cryobiotechnology of *Lilium* spp.

**DOI:** 10.1186/s13007-019-0506-9

**Published:** 2019-11-02

**Authors:** Jing-Wei Li, Xiao-Chen Zhang, Min-Rui Wang, Wen-Lu Bi, M. Faisal, Jaime A. Teixeira da Silva, Gayle M. Volk, Qiao-Chun Wang

**Affiliations:** 1 State Key Laboratory of Crop Stress Biology in Arid Region, College of Horticulture, Northwest A&F University, Yangling, 712100 Shaanxi People’s Republic of China; 2 College of Agronomy, Guizhou University, Guiyang, 550025 Guizhou People’s Republic of China; 3 Gosling Research Institute for Plant Preservation (GRIPP), Department of Plant Agriculture, University of Guelph, Bovey Bldg, Room 4227, Guelph, ON Canada; 4 Department of Botany & Microbiology, College of Science, King Saud University, P.O. Box 2455, Riyadh, 11451 Saudi Arabia; 5 P. O. Box 7, Miki-cho Post Office, Ikenobe 3011-2, Kagawa-ken 761-0799 Japan; 6 National Laboratory for Genetic Resources Preservation, 1111 S. Mason St, Fort Collins, CO 80521 USA

**Keywords:** Cryopreservation, Genetic stability, Lily, Pollen, Seeds, Shoot tips

## Abstract

*Lilium* is one of the most popular flower crops worldwide, and some species are also used as vegetables and medicines. The availability of and easy access to diverse *Lilium* genetic resources are essential for plant genetic improvements. Cryopreservation is currently considered as an ideal means for the long-term preservation of plant germplasm. Over the last two decades, great efforts have been exerted in studies of *Lilium* cryopreservation and progress has been made in the successful cryopreservation of pollen, seeds and shoot tips in *Lilium*. Genes that exist in *Lilium,* including those that regulate flower shape, color and size, and that are resistant to cold stress and diseases caused by fungi and viruses, provide a rich source of valuable genetic resources for breeding programs to create novel cultivars required by the global floriculture and ornamental markets. Successful cryopreservation of *Lilium* spp. is a way to preserve these valuable genes. The present study provides updated and comprehensive information about the development of techniques that have advanced *Lilium* cryopreservation. Further ideas are proposed to better direct future studies on *Lilium* cryobiotechnology.

## Highlights

- Availability of diverse genetic resources is essential for *Lilium* genetic improvements;
- Cryopreservation is an ideal means for the long-term preservation of plant germplasm;
- This review provides recent advances in the said field;
- Future prospects are proposed to direct further studies.

## Background

*Lilium,* a perennial bulb plant, includes approximately 100 species [1]. About 55 species and 32 varieties of *Lilium* are native to China [2], 21 *Lilium* species to North America, and 10 *Lilium* species to European and Caucasus [1].

*Lilium* is globally grown as one of the most important cut and pot flower and garden crops, mainly due to large, fragrant, multi-colored flowers with a long shelf-life [3, 4]. *Lilium* species and hybrids are also used as garden plants [3]. The bulbs of some *Lilium* species including *L. davidii* var. *unicolor, L. brownii* var. *viridulum* and *L. lancifolium* are widely consumed as vegetables [5] due to their high levels of protein, amino acids, minerals and dietary fiber [6, 7]. Other *Lilium* species such as *L*. *regale, L. concolor, L. pumilum, L. davidii* var. *unicolor* and *L. lancifolium* are traditional Chinese medicines [8], and they contain biochemical compounds such as alkaloids, polysaccharides, saponin and colchicine that have antioxidant activities [6, 9]. Lilies have been grown as vegetables and medicine for at least 2000 years in China [10].

A wide diversity of *Lilium* genetic resources has provided valuable genes for breeding of novel cultivars. There are more than 300 new registrations of *Lilium* cultivars each year and approximately 9500 cultivars in total globally [11]. There is a demand for new, novel lily cultivars from the production industry [4, 12, 13].

The availability of and easy access to diverse plant genetic resources are essential to provide the valuable genes necessary to develop novel cultivars. Extensive explorations and collections, heavy grazing, forest planting and urban expansions have threatened wild *Lilium* species [14, 15]. Global climate warming, which has increased the mean temperature of the Earth’s surface by approximately 0.8 °C from the early 1900s to 2011 [16], has further worsened the situation [15, 17]. *L. tsingtauense, L. polyphyllum, L. pomponium* and *L. maculatum* var. *bukosanense* are listed as endangered species in China [14], Italy [18], India [15] and Japan [19] respectively. As a result, it is necessary to preserve and protect *Lilium* genetic resources.

Cryopreservation is a method whereby plant genetic resources can be conserved in liquid nitrogen (LN, − 196 °C) or liquid nitrogen vapor (LNV, approx. − 165 to − 190 °C) [20–22]. Once stored in liquid nitrogen conditions, cellular divisions of the samples cease, and theoretically, plant materials can be maintained for extended lengths of time [20–23]. Once placed into long-term storage, less space and fewer resources are required to maintain secure collections, compared to field or in vitro collections [20–22]. Cryopreservation eliminates the risk of losing collections as a result of pathogen attacks and unexpected environmental conditions in field collections and reduces the risk of losing cultures by contamination and of genetic variations by repeated subcultures in in vitro collections [20–22].

This review provides updated and comprehensive information on the developments and advances in *Lilium* cryopreservation. In additional to preserving genetic resources, successful cryopreservation provides a method to conserve valuable genes in *Lilium* spp. that regulate flower shape, color and size, and that are resistant to cold stress and diseases caused by fungi and viruses. Prospects are proposed to direct further studies on *Lilium* cryobiotechnology.

### Cryopreservation

Bouman and De Klerk [24] were the first to cryopreserve shoot tips of *L. speciosum* with about 8% survival. Since then, various cryopreservation methods have been described for *Lilium* pollen [25], seeds [26–29] and shoot tips [4]. The major cryoprotocols available for *Lilium* include dehydration [26, 28, 29], encapsulation–dehydration [29, 30], encapsulation–vitrification [30], vitrification [30–33], and droplet-vitrification [34–38].

**Fig. 1:**
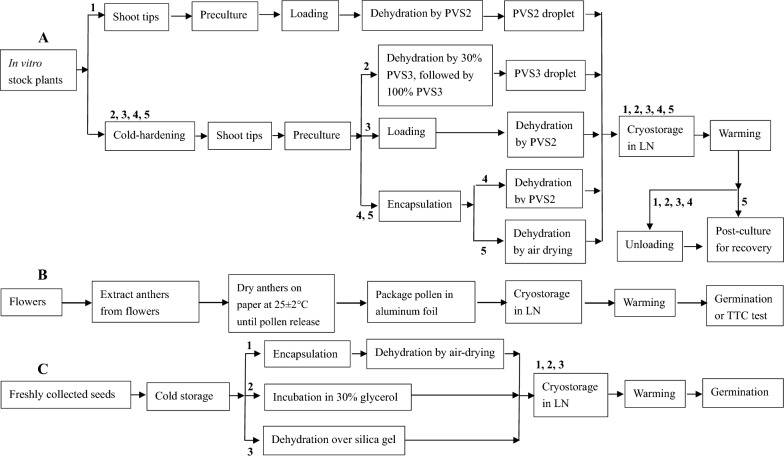
Major steps of *Lilium* cryopreservation for shoot tips (**A**), pollen (**B**) and seeds (**C**). Shoot tip cryopreservation by droplet-vitrification (1 in A [35]; 2 in A [34, 43]), vitrification (3 in A [30, 31]), encapsulation–vitrification (4 in A [30]), and encapsulation–dehydration (5 in A [30]). Pollen cryopreservation by dehydration (B [53]). Seed cryopreservation by encapsulation–dehydration (1 in C [29]), glycerol treatment (2 in C [29]) and dehydration (3 in C [29])

Major steps of *Lilium* cryopreservation for shoot tips, pollen and seeds are provided in Fig. 1.

### Shoot tips

A shoot tip refers to an apical dome and a few leaf primordia that are composed of undifferentiated cells capable of continuous cellular division and giving rise to a shoot [39]. For vegetatively propagated plants for which specific allelic combinations must be preserved, shoot tips are preferred over cell suspensions, seeds and embryogenic tissues for preservation of plant germplasm, because specific genotypes are conserved [20, 21]. Lilies are vegetatively propagated in the floriculture industry because specific cultivars must be maintained and therefore, shoot tips are often the preservation target for cryopreserving *Lilium* germplasm. A list of successful cryopreservation of *Lilium* shoot tips is presented in Table 1.

**Table 1:** A list of successful cryopreservation of shoot tips of *Lilium* spp.

Species or hybrids	No. of genotypes tested	Cryo-methods	Shoot regrowth (%)	Refs.
*L. japonicum*	1	Encapsulation–dehydration	88	[30]
*L. japonicum*	1	Encapsulation–vitrification	94	[30]
*L. japonicum*	1	Vitrification	92	[30]
*L. japonicum* and Oriental hybrids	1 for *Lilium japonicum* and 5 Oriental hybrids	Vitrification	83 for *Lilium japonicum* and 40–85 for five Oriental hybrids	[31]
*L. longiflorum*, *L. formosanum, L. henryi, L. auratum,* Oriental hybrids and Asiatic hybrids	2 cultivars for *L. longiflorum,* one for each of *L. formosanum, L. henryi, L. auratum,* 3 Oriental hybrids and 2 Asiatic hybrids	Vitrification	53–89 for *L. longiflorum,* 49–71 for *L. formosanum,* 5–19 for *L. henryi,* 51–66 for *L. auratum,* 53–93 for three Oriental hybrids and 53–89 for two Asiatic hybrids	[48]
Oriental hybrids ‘Siberia’	1	Vitrification	51 (survival). Shoot regrowth was not tested	[32]
*L. lancifolium*	1	Vitrification	95 (survival). Shoot regrowth was not tested	[49]
Oriental hybrid ‘Siberia’	1	Vitrification	72	[33]
*L. lancifolium, L.* × *longiflorum* and Oriental hybrid ‘Siberia’	3	Droplet-vitrification	67 for *L. *× *lancifolium* and 35 for *L*. × *longiflorum* and 60 for Oriental hybrid ‘Siberia’	[36]
*L. callosum* and Oriental hybrids	5	Droplet-vitrification	78 for *L*. callosum and 53–88 for the four Oriental hybrids	[34]
*Lilium* spp, Asiatic hybrid and Oriental hybrid	20 *Lilium* species, 1 Asiatic hybrid and 1 Oriental hybrid	Droplet-vitrification	52 for *L. amabile*, 67 for *L*. *auratum,* 43 for *L. bulbiferum,* 67 for *L. callosum,* 62 for *L. candidum,* 42 for *L. concolor, 85* for *L. davidii,* 41 for *L. distichum,* 48 for *L. formolongi*, 86 for *L. hansonii*, 38 for *L. henryi*,41 for *L. lancifolium,* 40 for *L. leichtlinii*, 69 for *L. leucanthum*, 43 for *L. longiflorum*, 41 for *L. pyrenaicum,* 83 for *L. regale*, 43 for *L. tsingtauense*, 41 *L. washingtonianum,* 43 for Asiatic hybrid, 73 for Oriental hybrid	[43]
*L. davidii* var. *unicolor*, *L.* × *formolongi, L. longiflorum *× Oriental ‘Triumphator’, Oriental hybrids and Asiatic hybrids	3 species, 2 Oriental hybrids and 2 Asiatic hybrids	Droplet-vitrification	80 for *L. davidii* var. *unicolor*, 53 for *L.* × *formolongi,* 43 for *L. longiflorum* × Oriental ‘Triumphator’, 73 for Asiatic hybrids ‘Elite’, 68 for Asiatic hybrids ‘Pollyanna’ and 88 for Oriental hybrid ‘Siberia’.	[35]
*L. martagon*	1	Droplet-vitrification	81–89	[38]

#### Droplet-vitrification

Vitrification refers to a physical process by which highly concentrated and viscous cryoprotectant mixtures form metastable glasses after rapidly cooling to LN, without the formation of ice crystals between and inside plant cells [40]. Vitrification cryopreservation is among the most often used methods for cryopreserving plant germplasm [21, 22, 41]. Droplet-vitrification combines the advantages of droplet freezing with vitrification, thus allowing samples to obtain rapid cooling and warming rates, increasing the survival of cryopreserved samples [41, 42]. Droplet-vitrification is considered to be a robust means for the cryopreservation of *Lilium* shoot tips [32, 34–36, 38, 43].

Chen et al. [36] described a droplet-vitrification for *Lilium* shoot tips. In their study, 2-months old in vitro lily stock shoots were cold-hardened at 4 °C for 1 week under a 16-h photoperiod of 35 µmol m^−2^ s^−1^ light intensity. Shoot tips excised from the cold-hardened shoots were precultured in liquid Murashige and Skoog [44] medium (MS) supplemented with 0.3 M sucrose for 2 days. Precultured shoot tips were loaded with a loading solution composed of MS supplemented with 2.0 M glycerol and 0. 4 M sucrose for 20–40 min at 22 °C, and exposed to plant vitrification solution 2 (PVS2) [45] for 90–120 min at 0 °C. PVS2 contains 30% (w/v) glycerol, 15% (w/v) ethylene glycol, 15% (w/v) dimethylsulfoxide (DMSO) and 0.4 M sucrose in MS (pH 5.8) [45]. Dehydrated shoot tips were transferred to 1.5-μL PVS2 droplets placed on sterile aluminum foil strips (5 × 30 mm), each strip carrying 5–6 shoot tips. The aluminum foil strips were folded to enclose the shoot tips and directly immersed into LN. For thawing, frozen aluminum foil strips were removed from LN and immediately placed in 1.2 M sucrose at room temperature for 15 min, followed by post-culture on a recovery medium for shoot regrowth. Recovery medium consisted of MS supplemented with 0.5 mg L^−1^ 6-benzylaminopurine (BA), 0.1 mg L^−1^ α-naphthaleneacetic acid (NAA), 0.3 mg L^−1^ gibberellic acid (GA_3_), 30 g L^−1^ sucrose and 7 g L^−1^ agar (pH, 5.7). The cultures were maintained in the dark for 2 weeks at 20 °C, and then cultured in a 16-h photoperiod of 35 µmol m^−2^ s^−1^ light intensity at 20 °C. Survival and shoot regrowth levels obtained by this droplet-vitrification cryoprotocol were 65% and 62% for Oriental hybrid ‘Siberia’, 84% and 68% for *L. lancifolium* and 43 and 35% for *L.* × *longiflorum,* respectively.

Yi et al. [34, 43] described a droplet-vitrification method for shoot tip cryopreservation of four Oriental hybrids and *L. callosum*. In their study, 2-week old in vitro stock cultures were cold-hardened for 7 days at 4 °C in a 16-h photoperiod of 35 µmol m^−2^ s^−1^ light intensity. Shoot tips were then excised from the cold-hardened in vitro stock cultures and precultured with 0.3 M sucrose overnight, followed by preculture with 0.7 M sucrose overnight under the same light conditions, as described for cold-hardening. Precultured shoot tips were exposed to 35% PVS3 [46] at 23 °C for 40–60 min, followed by exposure to full-strength PVS3 for 240 min, prior to direct immersion in LN. Exposure of shoot tips to 35% PVS3 produced the highest shoot regrowth in cryopreserved shoot tips: 87.5% for ‘Carmina’, 64% for ‘Crystal Light’, 53% for ‘Santander’ and 68% for ‘Marrero’ (61%), and 78% for *L. callosum* [34]. PVS3 contains 50% (v/v) glycerol and 50% (w/v) sucrose in MS [46]. PVS3 has been found to be less toxic to the cells than PVS2 and appeared to better protect plant tissues against osmotic stress, thus resulting in better survival of shoot tips after cryopreservation [47]. Droplet-vitrification has been applied to cryopreservation of 160 *Lilium* accessions at the National Academy of Agricultural Sciences in South Korea, including 20 species, one Asiatic hybrid and one Oriental hybrid [43]. The highest and lowest levels of shoot regrowth in cryopreserved shoot tips were 86% for *L. hansonii* and 38% for *L. henryi*, averaging at 56% for the total *Lilium* germplasm tested [43].

**Fig. 2:**
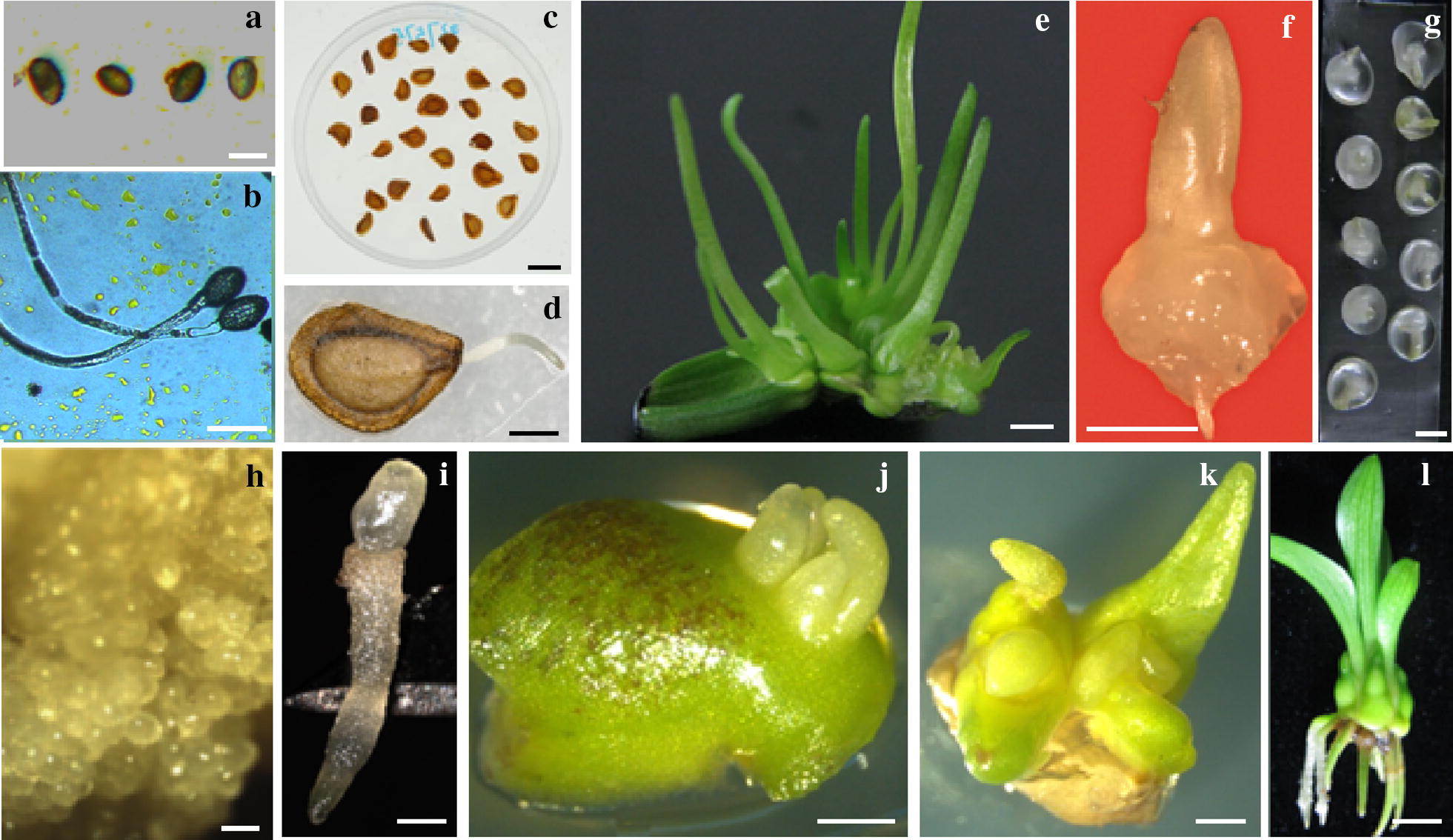
Cryopreservation of *Lilium* pollen (**a**, **b**), seeds (**c**, **d**) and shoot tips (**e**–**l**). Oriental hybrid ‘Siberia’ pollen released from anthers and used for cryopreservation (**a**) and germinating pollen (**b**) after cryoexposure (courtesy of Prof. Yan Liu for providing the photos). Surface-disinfected seeds of *Lilium brownie* used for cryopreservation (**c**) and germinating seeds (**d**) after dehydration cryopreservation (courtesy of Dr. Liang Lin for providing the photos). Four-weeks old adventitious buds regenerated from leaf segments of Oriental hybrid ‘Siberia’ (**e**). A shoot tip excised from the 4-weeks old adventitious buds in **d** (**f**). PVS2 droplets, each containing one shoot tip, on an aluminum foil strip (**g**). Somatic embryo-like structures regenerated from cryopreserved shoot tips of Oriental hybrid ‘Siberia’ after 4 weeks of post-thaw culture (**h**). A germinating somatic embryo developed from **h** after 4 weeks of culture on germinating medium (**i**). A small leaf square-bearing two adventitious buds of Oriental hybrid ‘Siberia’ after 4 weeks of induction (**j**). Shoot regrowth of cryopreserved small leaf square-bearing adventitious buds after 4 weeks of post-thaw culture (**k**). A plantlet regenerated from cryopreserved shoot tips of Oriental hybrid ‘Siberia’ after 4 weeks of culture on rooting medium (**l**). Bars in **a** and **b** = 20 µm; in **c** and **d** = 2 mm; in **e**–**l** = 1 mm

Cold-hardening (0–5 °C for 5–7 days) of in vitro stock shoots was necessary in many of the studies for successful cryopreservation of *Lilium* shoot tips [24, 30–32, 34, 36, 38, 43, 48]. Cold hardening requires a temperature-controlled growth chamber and is also time consuming. Yin et al. [35] described a droplet-vitrification cryopreservation for lily shoot tips in which cold-hardening of in vitro stock shoots was eliminated. In their study, adventitious buds were induced from leaf segments cultured on MS medium supplemented with 1 mg L^−1^ NAA and 0.5 mg L^−1^ thidiazuron (TDZ) in a 16-h photoperiod with a light intensity of 45 μmol m^−2^ s^−1^. Shoot tips (Fig. 2f) excised from 4-week-old adventitious shoots (Fig. 2e) were cultured on a basal medium (BM) composed of MS containing 30 g L^−1^ sucrose (pH, 5.7) for 1 day. Shoot tips were precultured on solid MS medium containing 0.5 M sucrose for 1 day and then loaded with a loading solution containing 0.4 M sucrose and 2 M glycerol for 20 min, followed by exposure to PVS2 for 4 h at 0 °C. Dehydrated shoot tips were transferred onto 2.5-μL PVS2 droplets on aluminum foil strips (Fig. 2g), prior to a direct immersion into LN for cryostorage. Frozen shoot tips were warmed in MS medium containing 1.2 M sucrose for 20 min at room temperature, followed by post-thaw culture for shoot regrowth on MS medium supplemented with 1 mg L^−1^ NAA and 0.2 mg L^−1^ TDZ (pH, 5.8). Cultures were placed at 23 ± 2 °C in the dark for 3 days and then transferred to the light to assess survival and shoot regrowth. Shoot regrowth levels ranged from 43% for Oriental hybrid ‘Triumphator’ to 88% for Oriental hybrid ‘Siberia’, with a mean shoot regrowth level of 67% across the six diverse *Lilium* genotypes tested.

#### Vitrification

Vitrification cryopreservation is among the most often used methods for cryopreserving plant germplasm [21, 22, 41].

Matsumoto et al. [31] described a method to cryopreserve *L. japonicum* shoot tips using vitrification techniques. In their study, single scale segments (5 mm × 5 mm) excised from in vitro stock shoots were cultured on a BM composed of MS containing 3% sucrose to induce adventitious buds. After 40 days of culture, scale segments with adventitious buds were cold-hardened at 0 °C for 28 days in a 12-h photoperiod of 60 µmol m^−2^ s^−l^ light intensity. Shoot tips excised from the cold-hardened adventitious buds were precultured on the basal medium composed of MS supplemented with 0.3 M sucrose at 25 °C for 1 day in the light. Precultured shoot tips were placed into 1.8-mL cryotubes and treated with a loading solution composed of 2 M glycerol and 0.4 M sucrose at 25 °C for 20 min. Loaded shoot tips were then exposed to PVS2 at 0 °C for 100–110 min or at 25 °C for 20 min, prior to direct immersion in LN for cryostorage. For warming, frozen cryotubes containing shoot tips were placed in a water bath at 40 °C for 2 min. PVS2 was drained from the cryotubes and replaced with 1.8 mL of 1.2 M sucrose solution for unloading at 25 °C for 20 min. Cryopreserved shoot tips were transferred onto sterile filter paper discs placed on the BM in a Petri dish, and placed in the light condition. After 1 day, the shoot tips were transferred onto fresh filter paper discs placed on the same medium and post-cultured in the same light condition for shoot regrowth. This vitrification protocol resulted in 40–85% of shoot regrowth in cryopreserved shoot tips of the five Oriental hybrids tested, and 83% in cryopreserved shoot tips of *L. japonicum* [31]. Vitrification was also successfully applied to shoot tip cryopreservation of four *Lilium* species [48], Oriental hybrids [32, 48], Asiatic hybrids [48], and *L. lancifolium* [49].

#### Encapsulation–vitrification and encapsulation–dehydration

Encapsulation–vitrification and encapsulation–dehydration are among the most popular cryoprocedures used for plant cryopreservation [21, 22, 41]. These two cryoprocedures both employ an encapsulation step whereby samples are encapsulated with 2.0–3.0% (w/v) calcium alginate into beads (3.0–4.0 mm in diameter). Beads are dehydrated by exposure to PVS2 in encapsulation–vitrification and by air-drying in encapsulation–dehydration.

Matsumoto and Sakai [30] reported encapsulation–vitrification and encapsulation–dehydration methods for cryopreserving *L. japonicum* shoot tips. In their study, in vitro stock shoots were cold-hardened and shoot tips were prepared, as described by [31], and then encapsulated with 2% Na-alginate solution in 0.1 M CaCl_2_ supplemented with 2 M glycerol and 0.4 M sucrose, to form beads (3 mm in diameter). For the encapsulation–vitrification method, the beads were exposed to PVS2 at 0 °C for 100 min, and then transferred into 2.0-mL cryotubes, each containing 10 beads and 0.7 mL PVS2, prior to direct immersion into LN for cryostorage. For regrowth assessment, frozen cryotubes containing shoot tips were warmed in a water bath at 40 °C for 2 min. PVS2 was drained from the cryotubes and replaced with 1.8 mL 1.2 M sucrose solution for unloading for 30 min at 25 °C. For the encapsulation–dehydration method, the beads were dehydrated over sterile filter paper placed in a desiccator containing 50 g of dried silica gel, which was sealed with Parafilm^®^, at 25 °C for 4 h, to reduce water content of the beads to about 26%. Dehydrated beads were transferred into 1.8-mL cryotubes and directly immerged in LN for cryostorage. Cryopreserved shoot tips were thawed as described in both encapsulation–vitrification, but without the unloading step using 1.2 M sucrose, as described above. In both the encapsulation–vitrification and encapsulation–dehydration methods, cryopreserved shoot tips were post-cultured for shoot regrowth, as described in vitrification by Matsumoto et al. [31]. This encapsulation–dehydration cryopreservation resulted in 88% shoot regrowth of *L. japonicum*.

### Pollen

In *Lilium*, hybrids are relatively easily obtained by crossing between species of the same section [50]. *Longiflorum* hybrids, Asiatic hybrids and Oriental hybrids are among the most valuable hybrids widely grown in the world [3, 50]. *Lilium* pollen viability differs between cultivars, genus and species, and usually lasts for several days [51]. Efficient preservation of pollen has potential for conserving plant genetic resources and for use in cross breeding [52].

Several laboratories in China have studied the cryopreservation of *Lilium* pollen [53–55]. Shi et al. [53] reported the cryopreservation of Oriental hybrids ‘Sorbonne’ and ‘Siberia’ pollen. Freshly harvested pollen was transferred into 1.5-mL cryotubes. The cryotubes were then placed in a 4 L desiccator filled with 1.0 kg silica gels for dehydration at 4 °C for 2 h, to reduce the water content of pollens to about 7.3–7.7% (fresh weight basis). Dehydrated pollen was placed at − 20 °C for 20 min, and then immerged in LN for cryostorage. Cryotubes containing cryopreserved pollen were warmed in a water bath set at 40 °C for 2 min and then in vitro germinated at 25 °C on a germination medium composed of 300 mg L^−1^ Ca(NO_3_)_2_ + 200 mg L^−1^ MgSO_4_ + 200 mg L^−1^ KNO_3_ + 200 mg L^−1^ H_3_BO_4_ + 20 g L^−1^ sucrose (pH 6.5) in the dark. Pollen germination levels were recorded after 12 h of culture. Results showed germination levels remained stable and high in pollen cryopreserved for different durations: 60% and 59% after 60 days, and 51% and 48% after 420 days, for ‘Sorbonne’ and ‘Siberia’, respectively.

The pollen of *L. concolor* var. *pulchelium* was successfully stored at – 80 °C [55]. Freshly harvested pollens were transferred onto paper tissue placed in a desiccator filled with silica gels for dehydration at room temperature for 8–12 h, to reduce the water content of pollen to 18–22%. Dehydrated pollen was stored directly in a refrigerator at − 80 °C or pre-frozen in LN and then transferred to a refrigerator at − 80 °C for storage. Stored pollen was warmed at 35 °C for 10 min and germinated in vitro. Ninety-one % of the pollen grains germinated after 240 days of storage, and more than 80% of pollen maintained their germination ability after 480 days of storage using these two methods [55].

Xu et al. [54] reported the successful cryopreservation of Oriental hybrid ‘Siberia’ pollen, using rapid freezing and PVS2 vitrification. In rapid cooling, pollen was transferred into cryotubes that were directly plunged into LN. Frozen cryotubes were removed from LN and rewarmed in a water bath set at 37 °C for 2 min. In vitrification, pollen were suspended in 2-mL microcentrifuge tubes, each containing 1.5 mL 0.01 M phosphate-buffered saline (PBS; 137 mM NaCl + 2.7 mM KCl + 4.3 mM Na_2_HPO_4_ + 1.4 mM KH_2_PO_4_; pH 7.2) to produce about 50 μL of packed pollen. Packed pollen was treated with a loading solution containing 0.4 M sucrose and 2 M glycerol for 20 min at 25 °C, followed by exposure to PVS2 for 50 min in an ice bath, prior to direct immersion in LN. Cryopreserved pollen were thawed, as described above for rapid cooling and then unloaded with 1.2 M sucrose at 25 °C for 10 min. Pollen viability, which was determined by the triphenyltetrazolium chloride reduction assay [56], was about 59% for rapid cooling and 70% for vitrification, both of which were significantly higher than 47% of the fresh (non-treated control) pollen.

Reactive oxidative species (ROS) are molecules that contain the element oxygen and are produced in plants under abiotic and biotic stress [57]. ROS-induced oxidative stress is closely related to pollen viability following cryopreservation [54, 58]. Viability of Oriental hybrid ‘Siberia’ pollen after rapid cooling (59%) and vitrification (70%) was significantly greater than that (47%) of the freshly collected pollen without any treatment [54]. Analysis of reactive oxygen species (ROS) found that ROS levels significantly increased during vitrification procedure. Similar results were reported for cryopreserved pollen in *Paeonia suffruticosa* [58]. However, results obtained in the same group showed that increased ROS levels resulted in decreased viability of cryopreserved pollen in *Jasminum nudiflorum*, *Malus spectabilis*, *Philadelphus pekinensis*, *Syringa oblate* and *Xanthoceras sorbifolium* [58]. These data indicate ROS levels affected viability of cryopreserved pollens and responses to ROS levels vary with plant species.

A list of successful cryopreservation of *Lilium* pollen is presented in Table 2 and Fig. 2a, b.

**Table 2:** A list of successful cryopreservation of *Lilium* pollens and seeds

Species or hybrids	Cryo-methods	Survival or germination (%)	Refs.
Pollens			
Oriental hybrid ‘Sorbonne’ and ‘Siberia’	Desiccation	60 and 59 after 60 days, and 51 and 48 after 420 days	[53]
Oriental hybrid ‘Siberia’	Rapid cooling	59	[54]
Oriental hybrid ‘Siberia’	Vitrification	70	[54]
*L*. concolor var. *pulchelium*	Desiccation + rapid cooling	21–92 (according to the conservation duration)	[55]
Seeds			
*L*. *ledebourii*	Encapsulation–dehydration	50	[27, 60]
*L. ledebourii*	Pregrowth-dehydration	75	[28]
*L*. *ledebourii*	Encapsulation–vitrification	10	[61]
*L. ledebourii*	Vitrification	97	[29]
	Desiccation	95	
	Encapsulation–dehydration	69	
	Glycerol pretreatment	98	
*L. martagon*	Dehydration	93–100	[64]

### Seeds

*Lilium* species, especially wild species, can be propagated by seeds. Therefore, seed cryopreservation can be considered an important strategy for the long-term preservation of *Lilium* germplasm. Studies on storage behavior of *Lilium* seeds are quite limited. A recent study [59] identified the seeds of *L. polyphyllum* had orthodox storage behavior. Seeds contain low moisture content after dispersal and can be dried to 5% or lower.

Two groups from Iran conducted a series of studies on cryopreservation of *L. ledebourii* seeds [27–29, 60–62] and developed various cryogenic protocols for this species, including encapsulation–dehydration [27, 29, 60], encapsulation–vitrification [61], pregrowth-desiccation [28, 62], vitrification and dessiccation and glycerol pretreatment [29]. A list of successful cryopreservation of *Lilium* seeds is presented in Table 2 and Fig. 2c, d.

Mohajeri et al. [29] used vitrification, encapsulation–vitrification, glycerol pretreatment and pregrowth-desiccation procedures to assess their effects on the cryopreservation of *L. ledebourii* seeds. Seeds that had been stored at 2–4 °C for 6 weeks without surface-disinfection were used for cryopreservation. In vitrification, seeds contained in 1.8-mL cryotubes were treated with a loading solution composed of 2 M glycerol and 0.4 M sucrose for 20 min at 22 °C and then exposed to pre-cold PVS2 for 20 min, followed by direct immersion in LN for cryostorage. In encapsulation–dehydration, seeds were encapsulated, as described by Kaviani et al. [61]. The beads were air-dried, as described by Kaviani et al. [61] for 3 h and transferred into 1.8-mL cryotubes, followed by direct immersion in LN for cryostorage. In glycerol pretreatment, seeds contained in 1.8-mL cryotubes were pretreated with 30% glycerol at 22 °C for 20 min. Cryotubes were then directly immerged in LN for cryostorage. In desiccation, seeds were placed in a desiccator containing 300 g silica gel for 21 h at 22 °C. The desiccated seeds were transferred into 1.8-mL cryotubes and directly plunged in LN for cryostorage. In all the four protocols tested, seeds cryopreserved in LN for 1 week were rapidly thawed in a water bath set at 40 for 2 min, and post-thaw cultured for germination on wet filter papers in 10-cm Petri dishes. The cultures were placed in a growth chamber at 22 °C with a 16 h photoperiod (light intensity not specified in the article) for 32 days. Seed germination percentage was about 98%, 97% and 95% in glycerol pretreatment, vitrification and desiccation, respectively, and much higher than 69% in encapsulation–dehydration. Although there were no significant differences in root length, shoot length was greater in seedlings recovered from cryopreserved seeds by glycerol pretreatment, vitrification and desiccation than in those by encapsulation–dehydration [29]. Glycerol treatment and desiccation avoid of use of toxic chemicals such as DSMO, which are required in vitrification, and easy to handle, and therefore, are recommended for cryopreservation of *Lilium* seeds [29].

Compared with the results obtained in the three cryoprocedures [29], as discussed above, only 10% survival levels of cryopreserved *L. ledebourii* seeds were obtained in encapsulation–vitrification [61], 50% in encapsulation–dehydration [27, 60, 61] and 75% in pregrowth-desiccation [26, 28].

Urbaniec-Kiepura and Bach [61] reported an improved pregrowth-desiccation protocol for cryopreservation of *L. martagon* seeds. In their study, seeds were cold-stored at 5 °C or 15 °C for 26 weeks. Following cold-storage, seeds were incubated on half-strength MS containing 0.75 M sucrose for 1 h, followed by desiccation by air-drying to reduce the moisture content of the seeds to 13.1%, prior to freezing in LN. With this protocol, 93–100% of seeds germinated following cryopreservation and seed germination time was shortened by about 34 days, compared with 41 days in non-cryopreserved seeds. A list of successful cryopreservation of *Lilium* seeds can be found in Table 2, and Fig. 2c, d.

### Comparison of cryopreservation efficiency

Matsumoto and Sakai [30], and Matsumoto et al. [31] compared effects of vitrification, encapsulation–vitrification and two encapsulation–dehydration procedures on shoot regrowth of cryopreserved shoot tips of *L*. *japonicum* ‘Japanese Lily’. In the first encapsulation–dehydration, the beads were precultured with 0.8 M sucrose, before dehydration, while in the second encapsulation–dehydration, the beads were precultured with 0.8 M sucrose and 1.0 M glycerol, before dehydration. Vitrification and encapsulation–vitrification produced 92–94% shoot regrowth levels, which were similar to 88% produced in the second encapsulation–dehydration procedure, but significantly higher than 67% produced in the first encapsulation–dehydration procedure. Matsumoto and Sakai [30], and Matsumoto et al. [31] attributed the improve shoot regrowth of cryopreserved shoot tips to glycerol treatment used in preculture by minimizing the cell membrane injury caused by dehydration. Responses to cryo-methods varied among *Lilium* genotypes [36]. Droplet-vitrification produced much higher shoot regrowth levels in Oriental hybrid ‘Siberia’ (65%) and *L. lancifolium* (84%) than vitrification in the corresponding lilies (35% and 36%). However, these two cryo-methods produced similar recovery levels (43% and 33%) in *L*. × *longiflorum* [36].

### Genetic stability

When samples are cryopreserved, cellular divisions halt, and theoretically, plant materials can be preserved for an extended length of time, while maintaining genetic stability [20–23, 63]. However, each of the necessary cryopreservation steps including preculture, cryoprotection, PVS exposure, thawing and post-thaw culture may cause physical or chemical stress, which subsequently may alter the genetic stability of plants recovered after cryopreservation [20–23, 63]. In addition, in vitro tissue culture techniques are also required for production of in vitro stock cultures and shoot regrowth after cryopreservation. In vitro tissue culture may induce genetic variations [64]. Therefore, the assessment of genetic integrity of regenerants recovered after cryopreservation is often performed [20–23, 34, 63].

To date, there have been only a few studies that evaluated morphology and assessed genetic integrity of the regenerants recovered after cryopreservation in *Lilium* [32, 35, 36, 43]. No differences in morphological traits including plant height, leaf length, leaf width and leaf color were observed between plants recovered from droplet-vitrification and vitrification cryopreservation of shoot tips and those of the control [32, 36]. No phenotypic differences were identified in three *Lilium* hybrids, *L. bolanderi* and *L. davidii* in plants recovered from shoot tips that were and were not exposed to LN, except the height of the LN-*exposed L. bolanderi* plants was slightly less than that of the controls [43]. Similar soluble protein and isoenzyme patterns were found in the plants recovered after shoot tips were cryopreserved by vitrification and the controls [34]. The use of inter-simple sequence repeat markers (ISSR) detected no differences in plants recovered from shoot tips that were and were not cryoexposed using droplet-vitrification techniques [35].

### Advances in improvements of explant production, cryopreservation efficiency and regenerative patterns of cryopreserved shoot tips

In many of the studies on the cryopreservation of *Lilium* shoot tips, adventitious buds induced from the bulb scales were cold-hardened at 0–5 °C for 5 days to 3 weeks and shoot tips excised from the cold-hardened stock cultures were used for cryopreservation [24, 30, 31, 34, 36, 47]. Cold-hardening of in vitro stock cultures requires a temperature-controlled growth chamber and is also time consuming. The droplet-vitrification protocol described by Yin et al. [35] eliminated cold-hardening of the in vitro stock cultures and at the same time produced high recovery levels (averaging at 67%) in six lily genotypes belonging to five *Lilium* species and hybrids.

In all studies so far reported for shoot tip cryopreservation, excision of shoot tips is a necessary step. This step requires skilled staff, and is the most time-consuming and labor-intensive in the whole cryoprocedures [65]. In addition, surgical excision of shoot tips from stock cultures may cause physical damage to and induce browning of explants. Recently, Pan et al. [33] reported the cryopreservation of small leaf squares-bearing adventitious buds of *L.* Oriental hybrid ‘Siberia’. In that study, adventitious buds were induced from leaf segments, as described by Yin et al. [35]. After 12 days of induction culture, small leaf squares (3 × 4 mm, Fig. 2j), each bearing at least one adventitious bud, were cut from leaf segments and subject to cryopreservation, according to Yin et al. [35]. With this procedure, 85% survival and 72% shoot regrowth were achieved following cryopreservation, with at least one shoot developed in each of the cryopreserved small leaf squares (Fig. 2k). Plantlets with a good root system formed after 4 weeks of culture on rooting medium (Fig. 2l). The use of small leaf squares bearing adventitious buds for cryopreservation eliminated the time-consuming and labor-intensive step of shoot tip excision, thus facilitating shoot tip cryopreservation.

In vitrification cryopreservation, cryotubes of 1.8–2.0 mL cryotubes are usually used. Working on vitrification for cryopreservation of *L. lancifolium* shoot tips, Xu et al. [49] found much higher survival levels (95%) were obtained when small cryotubes (200 μL) were used than those (75%) produced by large cryotubes (1.0 mL). Improved effects of small cryotubes on recovery of cryopreserved shoot tips are most likely due to faster cooling/warming rates produced in cryotubes with a smaller volume [45].

Somatic embryogenesis has potential applications to micropropagation and genetic transformation in *Lilium* [13, 66–68]. Establishment of somatic embryogenic tissues is time- and labor-consuming. Some lilies are still recalcitrant to somatic embryogenesis [66–68]. Once established, somatic embryogenic tissues need to be repeatedly subcultured to maintain their regenerative ability. Subculture has risks of contamination, thus resulting in total loss of the cultures, and regenerative ability of the embryogenic tissues decreases as times of subculture increase [67, 68]. Cryopreservation of somatic embryogenic tissues appeared to be difficult and to our knowledge no studies have been published in *Lilium*. Use of different post-culture media for recovery of shoot tips following droplet-vitrification as described by Yin et al. [35], Bi et al. [37] achieved three types of regenerants: (1) only embryo-like structures (Fig. 2h), (2) shoot regrowth with embryo-like structures, and (3) only shoot regrowth. The highest frequencies (≥ 75.0%) of total embryo-like structures were obtained from cryopreserved shoot tips post-cultured on the recovery medium containing 0.1 mg L^−1^ NAA and 0.1–0.2 mg L^−1^ kinetin (KT). The highest frequencies (≥ 25%) of total shoot regrowth were produced from cryopreserved shoot tips post-cultured on recovery medium containing 0.1 mg L^−1^ NAA and 0.05–0.4 mg L^−1^ KT. Frequencies of total embryo-like structures and shoot regrowth ranged from 45% to 90% and 25 to 53%, respectively, from six lily varieties belonging to five *Lilium* species or hybrids [37]. These embryo-like structures developed into somatic embryos and germinated (Fig. 2i) into whole plantlets when cultured on MS medium containing 90 g L^−1^ sucrose. This cryo-method provides alternative to cryopreservation of somatic embryogenic tissues in *Lilium*.

## Future prospects and conclusion

A number of valuable genes and transcription factors that regulate flower time, and flower shape, color and scent, as well as those resistant/tolerant to abiotic stresses have been reported in the genus *Lilium* [3, 50, 69–79]. For example, genes and transcription factors that regulate coloration and pigmentation were obtained in Asiatic hybrids [70–75] and *L. lancifolium* [76]. Genes encoding resistance to abiotic stresses such as cold, drought and salt were isolated from *L. lancifolium* and *L. regale* [78, 79]. Diseases induced by fungi such as *Fusarium* wilt and leaf blight [80, 81], and viruses such as cucumber mosaic virus (CMV), lily mottle virus (LMoV), lily symptomless virus (LSV) and tulip breaking virus (TBV) [80] cause severe damage to the lily industry. Genes encoding resistance to fungal and viral diseases have been found, including those resistant to *Fusarium* wilt in Asiatic hybrids [81] and *L. regale* [82–85], leaf blight in *L. regal* [86, 87], and TBV and LMoV in *Longiflorum* [72, 88, 89], Asiatic hybrids [73] and *L. regal* [74]. These valuable genes have been/are being used for breeding of novel cultivars resistant to abiotic and biotic stresses in classic and biotechnological strategies in *Lilium* [13, 72, 74–76, 85]. Cryopreservation of these valuable genes will certainly ensure supply genetic resources for breeding of novel lily cultivars in the future.

The droplet-vitrification method, which was developed by Panis et al. [41] for cryopreservation of the diverse genetic resources of the genera *Musa* and *Ensete* and has been widely used for cryopreservation of a huge number of plant species [20–22], was also proven to be the most applicable to a wide range of *Lilium* species, hybrids and cultivars [33–35], and should be tested to determine if they can be used to develop *Lilium* cryobanks for diverse genetic resources. It may be particularly valuable to cryopreserve Asiatic and Oriental hybrid lilies that contain key flower trait genes of interest and *L. regale* that is resistant to biotic and abiotic stress. Additional seed cryopreservation and longevity studies are also needed for both wild and cultivated species. Although several methods have been described for cryopreserving *Lilium* pollen [25, 53, 54], there was documentation of the use of cryopreserved pollen for making crosses and producing viable seeds. More studies that assess genetic stability in plants recovered after cryopreservation are needed to confirm that there are no genetic changes resulting from cryoexposure [35, 36, 68]. This additional research will aid in the development of *Lilium* cryobanks. Nevertheless, more studies are still needed to accelerate setting-up of cryo-bankings of diverse genetic resources in the genus *Lilium*.

At this time, there are no reports of the cryopreservation of *Lilium* somatic embryogenic tissue. Successful cryopreservation of somatic embryogenic tissues would facilitate the development of somatic embryogenesis applications in biotechnology programs in *Lilium*.

Virus diseases cause great economic losses in *Lilium* production [80]. *Lilium* elite cultivars used for flower production are vegetatively propagated and are therefore prone to virus infection [80]. Cryotherapy, a novel biotechnology for pathogen eradication [39], has been applied to 21 plant species and has successfully eradicated 28 viruses belonging to 18 genera of 8 families and one unassigned family [90]. Development of cryotherapy for production of virus-free plants would assist sustainable development of the *Lilium* industry.

## Data Availability

Not applicable.
